# Platelet rich fibrin and commercial sealants for dural closure in neurosurgery: An in vitro study

**DOI:** 10.1371/journal.pone.0319349

**Published:** 2025-04-01

**Authors:** Katerina Argiti, Mukesch J. Shah, Kevin Joseph, Vidhya M. Ravi, Angeliki Stathi, Florian Volz, Amir El Rahal, Daniel Strahnen, Jürgen Grauvogel, Roland Rölz, Ulrich Hubbe, Jürgen Beck, Ioannis Vasilikos

**Affiliations:** 1 Department of Neurosurgery, Medical Center, University of Freiburg, Faculty of Medicine, University of Freiburg, Freiburg, Germany; 2 Medical Faculty, University of Freiburg, Freiburg, Germany; 3 Laboratory of Experimental Brain & Spine Surgery (LENS), Medical Center, University of Freiburg, Faculty of Medicine, University of Freiburg, Freiburg, Germany; Arizona State University, UNITED STATES OF AMERICA

## Abstract

**Background:**

Watertight closure of the dura mater is essential after neurosurgical interventions to avoid complications such meningitis, intracranial hypotension and surgical site infections. In addition to conventional suturing techniques, various biomaterials have been proposed as sealants to minimize the risk of leakage. We previously reported that patient derived platelet-rich fibrin (PRF), an autologous biomaterial, significantly improves watertight dural closure when applied as an onlay over a standard running suture. To demonstrate the efficacy of this method, we aimed to compare this orthobiological approach with other commercially available sealants.

**Methods:**

We utilized an in vitro testing device that allowed for the assessment of watertight dura mater closure via hydrostatic testing. On our testing phantom using freshly harvested bovine dura maters, a standardized 20-mm incision was closed using a running suture, and the leak pressure was measured initially without (primary leak pressure) and subsequently with on lay augmentation (secondary leak pressure) using either PRF, fibrinogen- and thrombin-coated collagen patch (TachoSil®), collagen matrix graft (DuraGen®), Polyethylenglykol (PEG) and polyethylene glycol ester and trilysine amine hydrogel solution (DuraSeal®), polyethylene glycol, protein-reactive polyethylene glycol monomer coated collagen matrix (Hemopatch®) or polyethylenimine component autospray sealant (Adherus®).

**Results:**

The outcomes demonstrate that the usage of a dural onlay in addition to the standard running suture exhibited a substantial improvement in leak pressure compared to the running suture alone. Specifically, the median leak pressure was improved by 3.5 times, where the combined approach was able to withstand 38 cm H_2_O, whereas the running suture alone had a median leak pressure of 11 cm H_2_O. Upon evaluating the performance of the sealants, we identified two categories of dural sealants: a medium performance group (30 ± 2 cm H_2_O) comprised of Adherus® (31 cm H_2_O), DuraGen® (31 cm H_2_O), and Hemopatch® (28 cm H_2_O), and a high-performance group composed of DuraSeal® (45.5 cm H_2_O), and TachoSil® (41 cm H_2_O). Patient-derived PRF was able to withstand a max pressure of 45 cm H_2_O, falling into the high-performance group. Of all the sealants tested in this study, the PRF based patch demonstrated the lowest variance in leak pressure among all the tested sealants.

**Conclusions:**

Autologous platelet-rich fibrin in a two-step procedure displayed enhanced augmentation and reinforced watertight closure of the dura mater, exhibiting a four-fold increase in leak pressure compared to standard running suture alone. Among other commonly utilized commercial sealants, it ranked second with demonstrated consistency in its performance.

## Introduction

**Fig 1:**
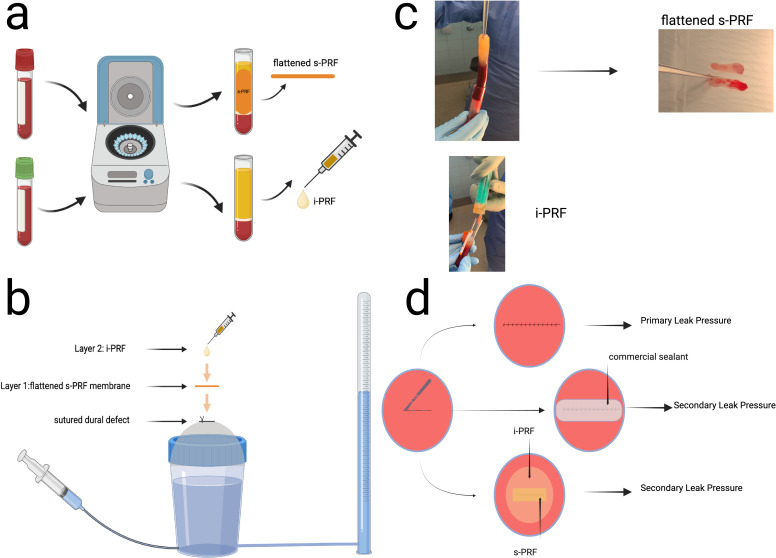
(a) Workflow depicting the preparation of the flattened solid platelet rich fibrin membrane (s-PRF) and the injectable platelet rich fibrin (iPRF). (b) Setup of PRF based dura repair. The standardized dural defect is initially treated with a running suture. Then the s-PRF membrane is applied as an on lay. In a second step i-PRF is instilled over the s-PRF membrane and the fibrin within starts forming a second layer of gelatinous membrane. (c) intraoperative view of s-PRF and i-PRF during preparation. (d) experimental workflow: after the dural incision is treated with the running suture, the hydrostatic pressure of the system gradually increases until a leak is observed, and the primary pressure is documented. After augmentation of the sutured dura with s-PRF and i-PRF, the same process is repeated, and the secondary leak pressure is documented.

Ensuring watertight closure of the dura mater is crucial in preventing complications following neurosurgical procedures [1,2]. Postoperative leaks of cerebrospinal fluid can lead to a range of conditions that are negatively correlated with successful outcomes, such as meningitis, wound-related problems, and intracranial hypotension [1,3]. Over the years, a variety of biomaterials have been suggested to augment the standard surgical practice of a running suture post neurosurgical interventions to function as sealants; however, there is a scarcity of high-quality evidence that compares and supports their efficacy [4]. Generally, there are two types of commercially available sealants. The first type, a synthetic absorbable sealant, is composed of PEG-based polymers. The second type, biological absorbable sealants, combines allogeneic or autogenic fibrinogens with allogeneic thrombin or collagen [4]. Both types of sealants can be found in liquid form or as patches. In addition to commercially available sealants, patient derived **p**latelet-**r**ich **f**ibrin (PRF) is a platelet concentrate that contains fibrin, leukocytes, cytokines, and growth factors that promote and accelerate natural wound healing and helps prevent local infections [5,6]. Depending on the application, PRF can be prepared with two different textures, either fluid (injectable PRF, i-PRF) or solid (solid PRF-s-PRF), according to the updated protocol of Choukroun and Ghanaati in 2018, after a single cycle of low-speed centrifugation. s-PRF is an elastic 3D fibrin matrix that can be shaped as needed to fit its use, whereas i-PRF is a fibrin matrix in an intermediate phase that has a high viscosity and gradually becomes gelatinous after atmospheric exposure [7–9] (Fig 1a, c). PRF is routinely utilized by several surgical specialties, including ophthalmology, orthopedic surgery, and vascular surgery, owing to its ability to adhere to tissues and act as a sealant [5,10–16].

We have previously reported on an in vitro hydrostatic assessment device, where watertight closure of dura mater could be hydrostatically assessed, that autologous platelet rich fibrin (PRF) applied as an i-PRF and s-PRF combination on lay could significantly reinforce a watertight dural closure in-vitro after the standard running suture [17]. In this study, we employed the same testing device and evaluated the efficacy of various materials as on lays for augmenting classical sutured dura closure under simulated conditions of increased intracranial pressure. Specifically, we compared collagen-bound fibrin sealant (TachoSil^®^), polyethylene glycol, polyethylenimine component autospray sealant (Adherus^®^), a collagen matrix graft (DuraGen^®^), protein-reactive polyethylene glycol monomer coated collagen matrix (Hemopatch^®^) and a Trilysinamin-based flexible hydrogel (DuraSeal®) to autologous platelet-rich fibrin (PRF). Among these, Adherus®, DuraGen®, and DuraSeal® have received approval from both the United States Food and Drug Administration (FDA) and the European Medicine Agency (EMA) for utilization as standalone or adjunct dural sealants in neurosurgery. TachoSil® has obtained FDA approval as an adjunct to hemostasis during cardiovascular, hepatic, and pulmonary surgery, as well as EMA approval for supportive treatment in surgery to enhance hemostasis, tissue sealing, and suture support in vascular surgery where standard techniques are insufficient. Additionally, Hemopatch® has received approval from both the FDA and EMA as a hemostatic patch for controlling hemorrhage in soft tissue and organ surgeries.

## Materials and methods

### Ethics statement

“This article does not contain any studies with human participants performed by any of the authors.”

“All applicable international, national, and institutional guidelines for the care and use of animals were followed.”

Our in vitro experiments were conducted in a manner that did not violate any provisions of the ARRIVE guidelines or the Declaration of Helsinki regarding animal studies. The bovine dura material used in the study was obtained from commercial meat processing, which conforms to the European regulatory standards. The blood utilized for the preparation of PRF was obtained from some of the participating researchers (healthy volunteers) after obtaining their oral and written consent. Following consultation with the University of Freiburg’s local ethics committee, it was determined that formal approval of the study was not required.

### Dura testing device and sample installation

We utilized the same hydrostatic testing simulator that was previously reported [17] (Fig 1b). Briefly, it consists of a transparent plastic container with a flat, cone-shaped bottom, with diameter of 4.5 cm. This main chamber was hydraulically interfaced with a measuring column that allowed us to assess changes in chamber pressure. The top of the chamber was secured with bovine dura samples (10 cm^2^), securely fitted with a plastic ring [17]. Each sealant was evaluated using 20 dura preparations obtained from 4 to 6-month-old bovine skulls sourced from the central meat processing unit in Freiburg. These preparations were refrigerated at 4°C until use, with a storage duration of less than one-week post-extraction.

### Preparation of solid and injectable PRF biomaterial

Platelet-rich fibrin (PRF) was prepared using blood samples obtained from donors who were free of any signs of infectious disease and did not use anticoagulants. A detailed protocol for PRF preparation in this study is described in our previous work [17](Fig 1a, c). It is based on the low centrifugation concept protocol initially described by Choukroun and Ghanaati [7], but with simplified categorization of the utilized PRF forms as solid PRF (s-PRF) for the solid membrane version of the leukocyte-platelet-rich fibrin arising from the centrifugation of blood in glass tubes (solid L-PRF) and as injectable PRF (i-PRF) for the liquid version of L-PRF (liquid L-PRF).

For each dura closure, we made use of 1 vial of s-PRF and 1 vial of i-PRF, each containing 10 ml of blood from healthy volunteers. The blood samples were centrifuged in a DUO centrifuge (1200 rpm for 8 min/177g, Process for PRF, Nice, France). This centrifuge features a fixed angle rotor with a 110 mm radius. Blood was centrifuged at 1200 rpm for 8 minutes (177 g) following the protocol [17]. Sterile 10 ml plastic tubes (Process for PRF, Nice, France) were used for i-PRF, and sterile 10 ml glass tubes (Process for PRF, Nice, France) for s-PRF. Blood was collected via a clinically approved butterfly method, and centrifugation started within 2-3 minutes post-collection. After centrifugation, tubes rested for 5 minutes. s-PRF was extracted and manually compressed from the glass tubes (Fig 1a, c), while i-PRF was aspirated into a 10 ml syringe from the plastic tubes [17]. The s-PRF membranes exhibited macroscopic similarities, and upon flattening, the resultant membrane was capable of completely covering the sutured dural defect in all measurements.

### Measurement of fluid leak pressure

#### Primary leak pressure.

After attaching the dura samples to the testing device, the watertight fixation and sample integrity were verified. The system was then filled with colored saline (dyed with: Evans Blue), ensuring that no air was trapped in the system (Fig 1b). No leakage was observed up to 70 cm H_2_O.

A straight 20 mm incision was made in the center of the dura using a No. 11 scalpel. The incision was then sutured using Premicron 4-0 running simple closure sutures, following the established standards for dural closure during neurosurgical procedures (as practiced at the University Clinic of Freiburg, Department of Neurosurgery). Suturing was performed with 2 to 3 throws per cm using 3 to 4 mm bites. Post suturing, the pressure in the chamber was slowly increased by the addition of dyed saline through the input valve. The hydrostatic pressure column was used to measure the increase in pressure. Primary leak pressure was recorded and defined as the first visible sign of a dural leak through the sutured dura. The pressure was then gradually decreased until no blue-dyed water remained above the sutured dura (Fig 1d).

#### Application of the dura sealants on the testing device.

In the second stage of the experiment, dura sealant was applied to the sutured incision covering the entire area, following the instructions of the manufacturer. The sealants used were TachoSil®, Adherus®, DuraGen®, DuraSeal®, and Hemopatch®, which were applied according to the manufacturer’s instructions provided in the user manuals. For PRF application, as previously discussed in our research [17], we adapted s-PRF into a membrane shape, which was then positioned on top of the sutured defect, fully covering it. In the following step, i-PRF was instilled drop-by-drop on top of the membrane, creating a thin layer that gradually solidified and developed into a biofilm (Fig 1d).

#### Secondary leak pressure.

In the second step, the pressure within the chamber was gradually increased according to the same algorithm, with the first visible sign of a dura leak through the sutured dura documented and noted as the secondary leak pressure. Throughout this phase, two observers monitored the chamber and pressure columns. To prevent internal errors caused by small leaks being masked by the PRF on lay, situations in which the pressure in the column dropped without a simultaneously observed leak in the chamber were documented as leaks. The entire process was captured and documented using a neurosurgery microscope (Pentero 900 from Zeiss Inc., Superlux® 330 light source with 2 ×  300 W xenon lamps) with a distance of 10 cm between the microscope and dura mater.

Throughout the aforementioned measurements and to prevent desiccation and subsequent contraction of the dural defect margins, the microscope light source intensity was set to 45%, and the sutured dural defect was promptly augmented with PRF following its preparation. The duration between surgical incision and closure was approximately 15 minutes.No discernible contraction of the dural defect margins was observed during the measurements.

### Statistical analysis

All statistical analysis was carried out in R computing environment. Reported values are medians ±  95% CI. Data distribution was assessed, and appropriate statistical methods were chosen, as indicated on associated plots. All recorded, analyzed data and code are publicly available and can be obtained at: kevinj24fr/PRF_comparison (github.com).

## Results

**Fig 2:**
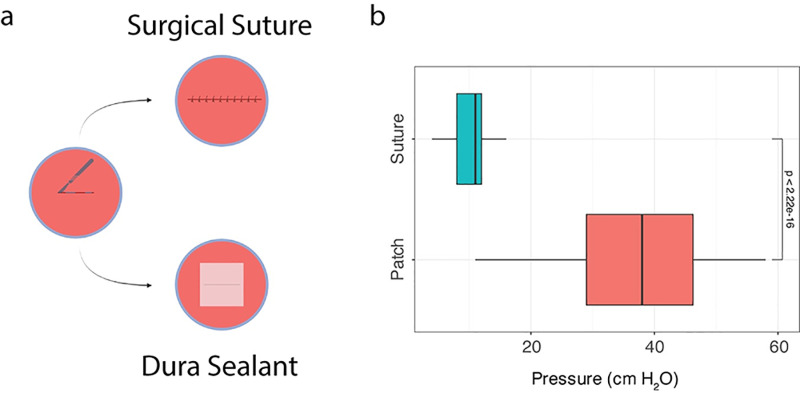
Experimental paradigm. (a) Experimental scheme used in this study. (b) Determination of effect size between sutures vs all sealants used in this study. The median pressure observed when using sutures alone was 11 ± 2.56 cm H_2_O vs 38 ± 11.4 cm H_2_O.

To ascertain whether a potentially clinically relevant and significant effect could be observed when utilizing a sealant, we conducted the experiment as delineated in Fig 2a. In brief, a linear incision was sutured using standard clinical practice to prevent cerebrospinal fluid leakage(Fig 2a). The utilization of any sealant enhanced the pressure retention capacity by 3.45-fold, from 11 ± 2.56 cm H_2_O to 38 ± 11.4 cm cm H_2_O, (Fig 2b).

**Fig 3:**
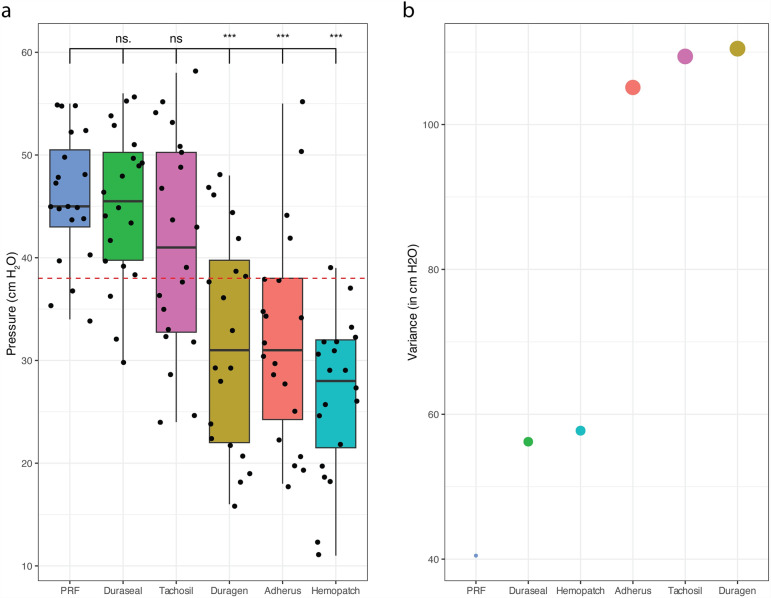
Comparison of the autologous PRF preparation vs commercially available sealants. (a) Leak pressure across all selants texted in this study. The dotted red line represents the median value of the leak pressure across all tested sealants. Observed values are represented as box plots, presented as median value ±  95% CI. p value represented as *=0.05, **=0.001, ***=0.0001 (b) Depiction of the variance in the measured values observed in each condition (cm H_2_O). The size of each of the points is proportional to the variance observed in the study.

We further explored the leak pressure observed when using various commercially available solutions vs the autologous PRF. We report that Duraseal and Tachosil do not exhibit statistically significant differences compared to the PRF combination (p > 0.05). Duragen, Adherus and Hemopatch exhibited significantly lower pressure retention capacity, (Fig 3a). In an effort to evaluate the reliability of the proposed PRF application in enhancing the dural sealing effect, we performed a variability analysis on our data. Among all other sealants interestingly, PRF exhibited the lowest variance of 40.484 ± 6.362 cm H_2_O, (Fig 3b).

## Discussion

Our results indicate that a two-layer orthobiologic on lay strategy that incorporates autologous platelet-rich fibrin in solid (s-PRF) and injectable (i-PRF) form as an on lay over the standard dura running suture could lead to a four-fold mechanical reinforcement of the watertight dural closure. Among the other examined commercially available dural on lay sealants PRF performed second after DuraSeal® although the difference of their median performance was not statistically significant. Additionally, the variation analysis revealed that PRF was more reliable than all examined commercial on lays. In a recent work of Coucke et al. 2024, a thorough cost-efficiency analysis revealed that PRF is significantly superior to other commercially available fibrin sealants [18]. Our simple and purely hydrostatic in-vitro testing design allowed us to reliably compare PRF with other commercial sealants based on a clinically relevant operative scenario setting, where a 20mm central dural defect is primarily closed with standard running closure suture. For each condition of our experiments, we performed 20 test-rounds to monitor how reliably each approach performs.

Previous studies have emphasized the significance of on lays in ensuring a tight closure of the dura in various types of surgeries, including those involving the spine [19], the skull [20], and the sphenoid sinus [21,22]. During surgery, the performance of the on lay is closely related to its ability to adhere to the dura mater and its elasticity, while after surgery, it is important for the on lay to become integrated with the healing dura in order to maintain a watertight seal. This is particularly important in cases where additional surgeries in the same area may be necessary. Therefore, an ideal on lay should possess not only excellent adhesive, mechanical, and water-tightness properties but also promote and support the healing process of the dura. Ebel et al. in 2022 on a comparable hydrostatic in vitro testing model, also evaluated the efficacy of various commercial sealants as an on lay to a standardized running suture closure of a 3 cm longitudinal dural defect [23]. This study demonstrated that DuraSeal®, a polyethylene glycol ester and trilysine amine solution, or TachoSil®, a fibrinogen- and thrombin-coated collagen patch, when used as an on lay with a 6-0 monofilament running suture, outperformed other commercial sealants with mean bursting pressures of 82.3 ±  12.72 cm H_2_O and 74.17 ±  12.64 cm H_2_O, respectively. These findings align with those of this study, which ranked DuraSeal® and TachoSil® among the top three dural on lays, along with PRF, in the group of high performing on lays. Unlike our experiments, Ebel et al. tested each on lay only six times, a limitation that we addressed by repeating the experiment 20 times for each condition to evaluate the sealants’ reliability. It is also important to mention that in our experiments we utilized for the running suture a 4-0 premicron suture, thus creating a thicker puncturing dural defect by each suture run, as this is the standard suture in our institution. Unlike earlier methods of preparing platelet-rich plasma, the second-generation PRF, which was initially introduced by Choukroun et al. in 2001, contains a greater number of leukocyte growth factors and key immune cytokines, and it also connects naturally with the surrounding tissues, promoting and accelerating all stages of wound healing [24–26]. In our study, the mechanical properties of PRF, along with its potential wound healing benefits, make it a promising candidate for further investigation as a dura-sealing onlay.

Besides it biomechanical and sealing properties, the main advantage of PRF is its autologous nature. Makoshi et al. in 2022 report a 24,5% of complications associated with the use of synthetic sealants in posterior fossa surgery [27]. Kuei-Lin Yeh et al. in 2022 additionally report on a cauda equina syndrome caused after application of a polyethylene glycol ester and trilysine amine hydrogel solution (DuraSeal^®^) in a lumbar spinal surgery case [28]. Others report on anaphylactic reactions after repetitive utilization of synthetic fibrin sealants [29,30].

The integrated biomechanical, sealing, and orthobiologic properties of PRF merit further clinical investigation. A recent case series study demonstrated its potential in a clinical setting, where PRF was used intraoperatively in a multi-layered reconstruction technique for the sellar floor after endonasal endoscopic transsphenoidal surgery. The study found that PRF in solid and injectable forms was easily utilized in the operating theater, effectively prevented cerebrospinal fluid leaks was safe and effective up to 12 months post-surgery [31].

The present study has certain limitations that prevent clear recommendations from being made regarding the use of a specific material or technique for dural closure. Firstly, bovine dura, rather than human dura, was utilized due to the availability of the former. It should be noted that bovine dura exhibits a similar tissue texture to human dura, as previously described in the literature [32]. Secondly, our in vitro model did not simulate various factors that exist in vivo, such as body temperature, the presence of blood and CSF, and pulsation. Additionally, it should be mentioned that at the end of surgery, sutures alone may not be sufficient to close the dura adequately, and therefore, additional sealants or dural substitutes may be used to cover and seal the small openings. Our in vitro model did not account for this possibility, as a standardized primary closure was always performed. Hence, the results obtained from our in vitro model cannot directly be applied to in vivo conditions. Third, in addition to the materials and techniques used, there are various other factors that can influence the development of postoperative cerebrospinal fluid (CSF) leakage. These factors may include the size and location of the dural opening and the surgeon’s skill and precision. Moreover, conducting a systematic evaluation like our in vitro trial can be challenging to replicate in in vivo studies. Fourth, it is important to note that our experiments were performed in a controlled in vitro environment and do not necessarily reflect the short-term or long-term incidence of CSF leakage in vivo. Finally, our in vitro model is a novel development and has not yet been fully validated. While our study included 20 experiments per test series, the sample size may be too small and further testing in larger sample sizes is needed to fully assess the reliability of our experimental design.

Another constraint of our experimental design is the flat nature of the dura mater layer, which enabled optimal use of i-PRF. Due to the time required for i-PRF to solidify, further investigation is needed for clinical applications on vertical or sloping dura mater surfaces. Our proposed method is well-suited for spinal surgery, where patients are typically in a prone position. In supratentorial cranial surgery, adjusting the operating table during the procedure may help ensure proper application of i-PRF. Our results suggest that PRF can efficiently augment the classical dural closure and its autologous nature, coupled with its simplicity in preparation and cost-efficiency, merits additional investigation in clinical settings.
